# Hyperspectal imaging technology for phenotyping iron and boron deficiency in *Brassica napus* under greenhouse conditions

**DOI:** 10.3389/fpls.2024.1351301

**Published:** 2024-05-24

**Authors:** Hui Li, Long Wan, Chengsong Li, Lihong Wang, Shiping Zhu, Xinping Chen, Pei Wang

**Affiliations:** ^1^ College of Engineering and Technology, Key Laboratory of Agricultural Equipment for Hilly and Mountain Areas, Southwest University, Chongqing, China; ^2^ Key Laboratory of Modern Agricultural Equipment and Technology (Jiangsu University), Ministry of Education, School of Agricultural Engineering, Jiangsu University, Zhenjiang, China; ^3^ National Citrus Engineering Research Center, Chinese Academy of Agricultural Sciences & Southwest University, Chongqing, China; ^4^ Interdisciplinary Research Center for Agriculture Green Development in Yangtze River Basin, College of Resources and Environment, Southwest University, Chongqing, China; ^5^ Institute of Urban Agriculture, Chinese Academy of Agricultural Sciences, Chengdu, China

**Keywords:** deficiency identification, rapeseed, iron and boron, hyperspectral imaging, MSC-CARS-LDA

## Abstract

**Introduction:**

The micronutrient deficiency of iron and boron is a common issue affecting the growth of rapeseed (*Brassica napus*). In this study, a non-destructive diagnosis method for iron and boron deficiency in *Brassica napus* (genotype: Zhongshuang 11) using hyperspectral imaging technology was established.

**Methods:**

The recognition accuracy was compared using the Fisher Linear Discriminant Analysis (LDA) and Support Vector Machine (SVM) recognition models. Recognition results showed that Multiple Scattering Correction (MSC) could be applied for the full band hyperspectral data processing, while the LDA models presented better performance on establishing the leaf iron and boron deficiency symptom recognition than the SVM models.

**Results:**

The recognition accuracy of the training set reached 96.67%, and the recognition rate of the prediction set could be 91.67%. To improve the model accuracy, the Competitive Adaptive Reweighted Sampling algorithm (CARS) was added to construct the MSC-CARS-LDA model. 33 featured wavelengths were selected via CARS. The recognition accuracy of the MSC-CARS-LDA training set was 100%, while the recognition accuracy of the MSC-CARS-LDA prediction set was 95.00%.

**Discussion:**

This study indicates that, it is capable to identify the iron and boron deficiency in rapeseed using hyperspectral imaging technology.

## 1 Introduction

Rapeseed, rich in oil and protein, is an important oil crop and industrial raw material, as well as a potential bioenergy crop overall the world (Liu et al., 2019). According to the Rapeseed Explorer of USDA, the global production of rapeseed has reached 87.103 million metric tons, which producing 31.8 million metric rapeseed oil (USDA, 2024). With various useful compounds of fatty acids, vitamins and proteins, rapeseed oil ranks as the third most popular vegetable oil after oil palm and soybean (Friedt et al., 2018). To better manage the fertilizer supply during rapeseed cultivation, it is essential to monitoring the micronutrients status of the plants.

Appropriate application content of fertilizers will not only benefit the absorption and utilization of nutrients by the crop plants, but also contribute to plants stress tolerance (Hasanuzzaman et al., 2018; Thor, 2019). On the contrary, lacking essential nutrients could inhibit the growth of the plants, which would directly lead to the negative effect on rapeseed quality or yield (Agren et al., 2012; Johnson et al., 2022). Real-time, fast, and accurate monitoring of nutrient content would provide guidance for reasonable fertilization to increase the crop quality and production in any specific regions (Brown et al., 2022; Tian et al., 2024). Therefore, monitoring the nutrient content of plants is an important aspect in crop cultivation and management.

As two of the essential micronutrients, boron and iron play important roles in the growth and reproduction of rapeseed, especially in the southwestern region of China. Boron participates in promoting the transport of carbohydrates *in vivo* plants which will accelerate the growth of apical shoots and meristem. It is also conducive to the development of plant flower organs (Kalaji et al., 2018; Li et al., 2020). When the rapeseed plant is in deficiency of boron, the transportation of assimilation products *in vivo* plant could be interrupted. As a result, a large amount of starch would accumulate in the leaves and petioles. Furtherly, the greatly increase of phenolic compounds content would lead to necrosis of plant apical buds. Thus, the main manifestation of boron deficiency in rapeseed is the inhibition of apical buds, which would interrupt the growth of roots and shoots, ultimately leading to the issue of “blooming but not setting fruit” of rapeseed plants. Iron acts as an activator of some enzymes or enzyme cofactors in the synthesis of chlorophyll. It would indirectly affect the production process of chlorophyll, while playing an important role in electron transfer chain in various biochemical reactions *in vivo* plants (Takano et al., 2008; Pavlovic et al., 2021). The main manifestation of iron deficiency in rapeseed is the chlorosis and yellowing between leaf veins while the leaf veins themselves remain green, especially in the top fresh leaves (Takano et al., 2008; Merchant, 2010).

Phenotyping technology with optical sensors such as RGB camera, chlorophyll fluorescence sensors, and particularly the spectral imaging system, has been widely applied in monitoring various biotic stresses for crops. With UAV-based RGB and multi-spectral sensors, salinity stress phenotyping has been realized in tomato and quinoa plants (Johansen et al., 2019; Jiang et al., 2022). The study results provided insight into the effects of salt stress on plant area, growth and condition. Optical information like chlorophyll fluorescence can also be measured for photosystem status evaluation such as investigating herbicide stresses in soybean plants (Li et al., 2018). Meanwhile, phenotyping of stresses from over or deficient macronutrients such as nitrogen, phosphorus, and potassium have also been successfully tested in many studies using hyperspectral imaging technology (Jiang et al., 2015; Tmušić et al., 2020). However, at present, the deficiency of the micronutrients iron and boron in crops is mainly evaluated using artificial vision and empirical morphological diagnostic methods which could only be made when obvious stress symptoms have appeared, and the specific fertilization may be missed for the suitable application time window.

The objectives of this study were to, (1) investigate if it was possible to differentiate the iron and boron deficiency symptoms in rapeseed from healthy plants at early growth stage using spectral imaging technology; (2) optimize the spectral diagnostic model for a high classification accuracy. The results will provide support for the nutritional diagnosis of iron and boron content in rapeseed fields using UAV-based sensing systems and even for the potential detection of vegetation deficiency symptoms via the space-based remote sensing satellites.

## 2 Materials and methods

### 2.1 Plant materials

The cultivar of tested rapeseed (*Brassica napus* L.) in this study is Zhongshuang 11 (ZS11, Beijing, Chinese Academy of Agricultural Sciences), which is widely grown in the Yangtze River basin. The plants were grown in 380 mm×300 mm pots with soilless hydroponic incubator with six plants per pot. All the plants were cultivated in a greenhouse of Southwest University in Chongqing, China.

The nutrient deficient plants were cultivated as the methodology described by Han et al. (2016), in which the ZS11 genotype was also cultivated as the tested plants. The rapeseed seeds were germinated in distilled water. After germination, the seedlings were transferred to a plastic net floating on the half strength modified Hoagland solution (**Table 1**). Normally growth seedlings were selected for next cultivation steps of the tests. Seedlings for control treatment were kept in the half strength modified Hoagland solution with boron concentration of 20 μmol L^-1^ (H_3_BO_3_) and iron concentration of 80 μmol L^-1^ (C_10_H_12_FeN_2_NaO_8_, EDTA-Fe), which were dramatically lower than the element concentration in the Chinese State Standard of foliar microelement fertilizer (State Administration for Marker Regulation of the People’s Republic of China & Standardization Administration of the People’s Republic of China, 2020). The seedlings for nutrient deficiency treatments were then transferred to solution with iron or boron in lower concentration. Boron deficiency plants was transferred to the solution with boron concentration of 0.5 μmol L^-1^, while the iron deficiency plants were treated with iron concentration of 1 μmol L^-1^. The other nutrients of both micronutrients deficient solution were kept in same concentration as the half strength modified Hoagland solution. The solution in all treatments was replaced every two days.

**Table 1:** Modified 1/2 Hoagland complete nutrient solution formula.

Chemical	Molecular Weight	Concentration (10–^3^ mol L^-1^)
Ca(NO_3_)_2_·4H_2_O	236.15	2500
KNO_3_	101.1	2500
NH_4_NO_3_	80.04	1000
K_2_SO_4_	174.26	250
MgSO_4_·7H_2_O	246.47	1000
KH_2_PO_4_	136.09	500
DETA-Fe	376.05	80
H_3_BO_3_	61.83	20
MnCI_2_·4H_2_O	197.91	4.5
ZnSO_4_·7H_2_O	287.54	0.3
CuSO_4_·5H_2_O	249.68	0.16
(NH4)_6_Mo_7_O_24_·4H_2_O	1235.86	0.16

Germination treatment was applied to full and consistent ZS11 seeds. The seeds were soaked in distilled water for 20 minutes and disinfected with 5% NaClO solution for 20 minutes. After rinsed with distilled water repeatedly for 5–6 times, the seeds were put on gauze soaked in 1/4 strength Hoagland solution for seedling cultivation.

Seedlings with uniform growth stage were selected for transplant. The plants were transferred to plastic hydroponic tanks containing nutrient solutions (1/4 strength Hoagland solution was used for cultivation in the first week after transplanting, half strength Hoagland solution was used for cultivation since the second week after transplanting, solution for nutrient deficiency treatments were applied since the third week after transplanting). Four biological replicates were applied for each treatment with 72 plants in total. The plants were set with a Randomized Block Design. The experiment was repeated twice in 3^rd^ March to 14^th^ June and 7^th^ September to 12^th^ December in 2023.

### 2.2 Hyperspectral imaging system

**Figure 1:**
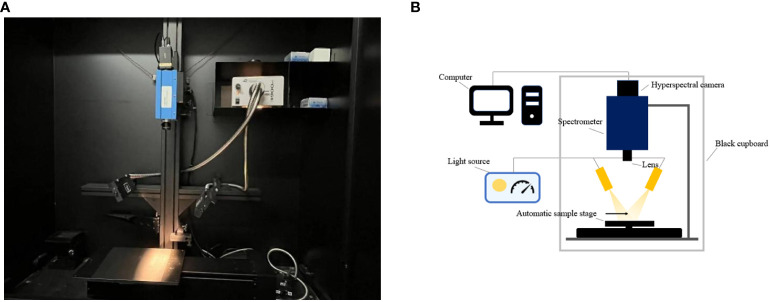
Hyperspectral imaging system **(A)** physical drawing; **(B)** architecture diagram.

The physical and architectural diagrams of the hyperspectral imaging system are shown in **Figures 1A, B**, respectively. The main hardware includes a hyperspectral camera (Raptor EM285CL, Raptor Photonics Led., UK), a spectrometer (Impector V10E, Measuring wavelength range 364~1025 nm, Spectral resolution 2.8 nm, Specim, Spectral Imaging Ltd., Finland), a zoom lens, a 150 W halogen adjustable light source, a linear photoconductor, a stepping motor mobile platform, a computer, etc. The whole set of devices is placed in the black box except the computer. The main software installed on the computer includes Spectral image, an image acquisition software provided by Wuling Optics (Taiwan, China), and HIS Analyzer, an image analysis software.

This device was fixed on the top of a black box in a darkroom. Each plant was moved to the measuring platform out from the hydroponic incubator. The window of the black box was closed during measurement.

### 2.3 Data collection and calibration

After 28 days of transplanting, hyperspectral images of rapeseed leaves were collected uniformly. To ensure the representativeness of the collected data, all samples were placed horizontally under the same conditions for imaging. After pre-testing, it was ultimately determined that the exposure time of the hyperspectral imaging system camera was 48 ms, the working distance from the lens to the sample was 480 mm, and the moving platform speed was 1.12 mm s^-1^.

Black and white board correction was performed on the hyperspectral image data of each sample in the image analysis software HIS Analyzer. The correction formula is as follows:


$$\text{R}=\left({\text{R}}_{s}-{\text{R}}_{D}\right)/\left({\text{R}}_{w}-{\text{R}}_{D}\right)$$

where, R is the relative reflection density of the leaves, ${\text{R}}_{s}$ is the reflection density of the original image of the sample, ${\text{R}}_{w}$ is the reflection density of all the white calibration image, and ${\text{R}}_{\text{D}}$ is the reflection density of the all black calibration image. Black and white correction is used to eliminate the influence of camera dark current, while converting the spectral values of the original hyperspectral image into reflectance.

### 2.4 Data preprocessing

**Figure 2:**
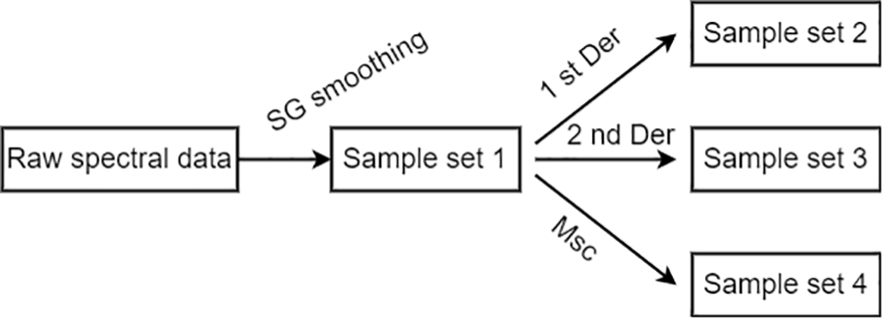
Flow chart of spectral data preprocessing.

Due to the influence of instruments, image acquisition background, environmental lighting conditions, and other factors, there would be noise, spectral baseline drift, and translation in the obtained spectral data. To eliminate these adverse effects on classification modeling, preprocessing of spectral data is necessary. After preliminary experiments, normalization, SG convolutional smoothing, spectral differentiation, and Multiple Scattering Correction (MSC) were selected for the spectra preprocess of the leaf samples after smoothing. The preprocessing procedure is shown in **Figure 2**. Four types of the spectral data obtained after preprocessing are spectral sample sets 1–4, which are abbreviated as RAW, 1st Der, MSC, and 2nd Der in the following text.

**Figure 3:**
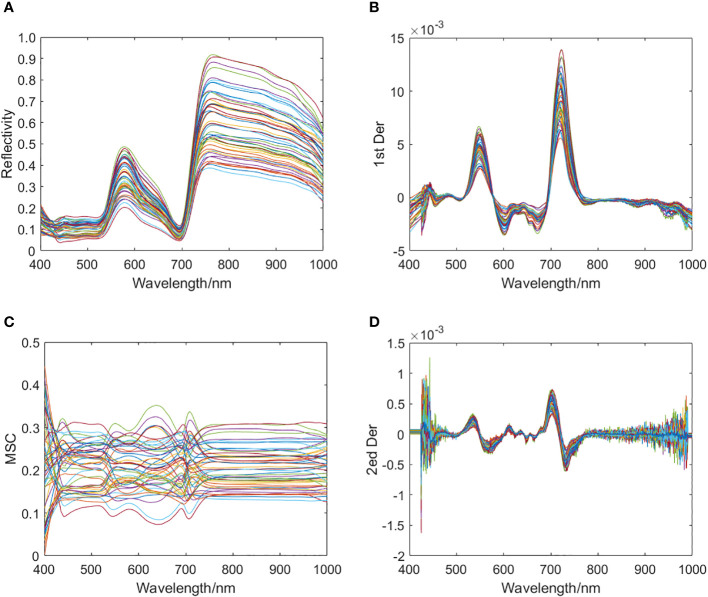
Four spectral samples obtained from preprocessing spectral data of some healthy rapeseed leaves. **(A)** Sample set 1: RAW; **(B)** Sample set 2: 1st Der; **(C)** Sample set 3: MSC; **(D)** Sample set 4: 2nd Der.

**Figure 3** shows four spectral samples obtained from the pre-processed spectral data of some healthy rapeseed leaves.

### 2.5 Band screening

Hyperspectral data often has hundreds or even thousands of wavelength points, which not only provides rich information about samples but also poses challenges for computer storage, transmission, and data processing (Arnon and Hoagland, 1938). When extracting spectral dimension information from hyperspectral data for modeling, using full band spectral information to establish the model will bring various negative impacts to the model due to the presence of uninformative variables in the data (Gruber et al., 2013). The dimension reduction algorithm can select the wavelength variables that are more meaningful to the classification results from the full wavelength range and eliminate redundant wavelengths. It could improve the prediction accuracy and modeling calculation efficiency of the model, as well as reducing the overfitting of the model and improve the generalization ability of the model (Arnon and Hoagland, 1938; Gruber et al., 2013; Khan et al., 2018).

#### 2.5.1 Continuous projection algorithm

Successive Projections Algorithm (SPA) is a forward variable selection algorithm, which uses vector projection analysis to select the combination of many variables with the smallest collinearity. In some studies on plant spectral feature classification and regression models, continuous projection algorithms are often applied in the dimensionality reduction process of hyperspectral data, which can play a good role in improving model operation efficiency and recognition accuracy (Belgiu and Drăguţ, 2016).

#### 2.5.2 Competitive adaptive reweighting algorithm

The Competitive Adaptive Reweighted Sampling (CARS) algorithm has also been widely applied in the recognition of plant spectral features. CARS uses the Monte Carlo sampling principle to select sample subsets for modeling, and to evaluate the importance of variables through the regression coefficients of the sub models. In each iteration, dimensionality reduction is achieved by removing variables with smaller mean regression coefficients through Exponential Decreasing Function (EDF) and Adaptive Reweighted Sampling (ARS) (Lorente et al., 2012).

### 2.6 Classification model

Linear Discriminant Analysis (LDA), also known as Fisher linear discriminant analysis, is a classic algorithm for pattern recognition and is widely used in multi class classification problems. Using LDA can maximize the inter class scatter matrix of the projected pattern samples and minimize the intra class scatter matrix, ensuring that the projected pattern samples have the minimum intra class distance and maximum inter class distance in a new space. Its essence lies in finding a subspace. It enables better separation of various categories in this subspace, which means that patterns have the best separability in that space (Zhang et al., 2022).

Support Vector Machine (SVM) is a supervised pattern recognition method. The original spectral data is mapped to a high-dimensional feature space, and an optimal classification hyperplane is constructed to maximize the distance between the support vectors of various samples and this hyperplane. SVM can be used for linear and nonlinear multivariate analysis problems, and the support vector can be solved by using linear equations instead of Quadratic programming. By selecting appropriate kernel functions to ensure the speed and efficiency of modeling while implementing nonlinear mapping (Yuan et al., 2020), this experiment uses Radial Basis Function (RBF) as kernel functions.

## 3 Results

### 3.1 Spectral features of nutrient deficient leaves

**Figure 4:**
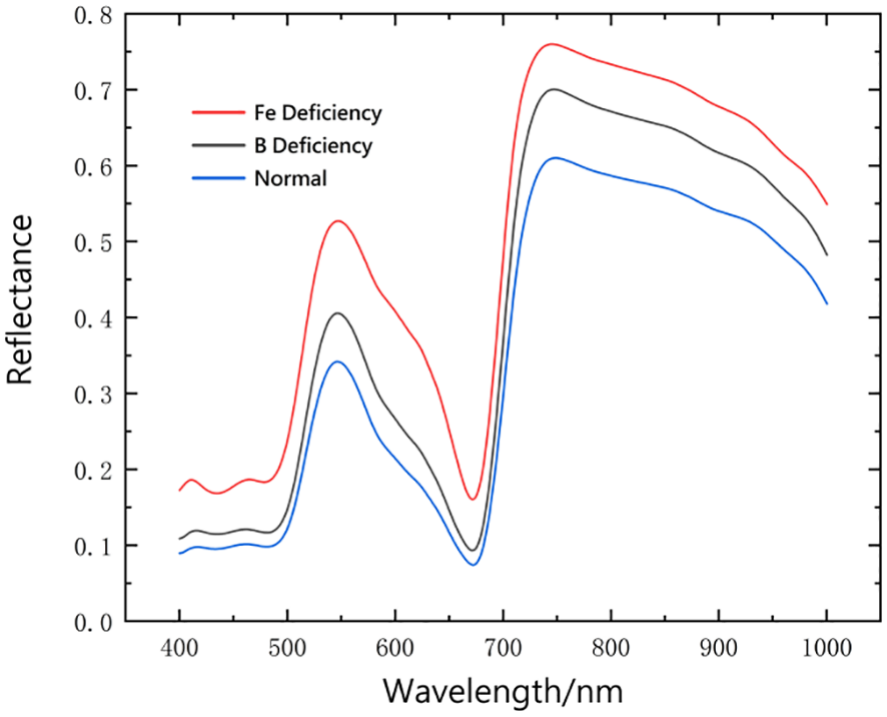
Comparison of average spectra between normal leaves and iron and boron deficient in *Brassica napus* leaves.

From the average spectra of the collected leaves of rapeseed plants (**Figure 4**), it presented that overall waveform of the spectral reflection curve in the wavelength range of 400–1000 nm was similar between normal plants and iron or boron deficient plants.

### 3.2 Deficiency recognition and classification model based on full band information

After preprocessing the 400–1000 nm full band spectral data using three preprocessing methods, known as spectral first order differential, spectral second order differential and MSC. LDA discriminative model and SVM discriminative model for identifying iron deficiency, boron deficiency and normal leaves were established respectively. LDA discriminative model is a typical Fisher linear discriminant analysis in Matlab Toolbox. When using SVM to build a discriminative model, the kernel function used when using SVM to build a discriminative model is the radial basis function (RBF) kernel function:


$$K(X_{i},X_{j})=\text{exp}{\left(-\gamma ∥X_{i}-X_{j}∥\right)}^{2}$$

In the SVM modeling, the Penalty coefficient y was set as 100, and the kernel width σ was set as 0.1. The discrimination results of each model were shown in **Table 2**.

**Table 2:** Discrimination results of LDA and SVM discrimination models under different spectral pretreatment.

Model	Preprocessing method	Training set		Testing set	
		Nc/Nt	Accuracy	Nc/Nt	Accuracy
SVM	RAW	81/90	90.00%	47/60	78.33%
	1st Der	78/90	86.67%	39/60	65.00%
	MSC	84/90	93.33%	52/60	86.67%
	2nd Der	83/90	92.22%	51/60	85.00%
LDA	RAW	79/90	87.77%	49/60	81.66%
	1st Der	81/90	90.00%	46/60	76.67%
	MSC	87/90	96.67%	55/60	91.67%
	2nd Der	84/90	93.33%	47/60	78.33%

“Nc” represents the correct discriminant number of the tested samples; “Nt” represents the total number of tested samples.

Comparing the discrimination accuracy of the two models, it presented that the LDA model had better overall discrimination performance than the SVM model. However, when using the LDA model to model the rapeseed leaf spectral dataset, the most suitable preprocessing method was MSC. When using the SVM model, the two preprocessing methods MSC and 2nd Der had better results.

**Figure 5:**
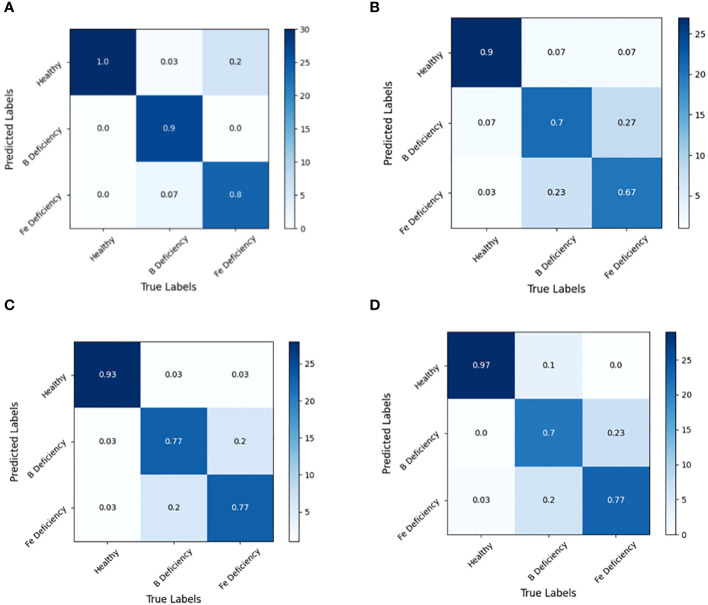
Discrimination results based on SVM models under different spectral pretreatments (Modeling set) for iron and boron deficiency in *Brassica napus*. **(A)** RAW, **(B)** 1st Der, **(C)** MSC, **(D)** 2nd Der.

**Figure 6:**
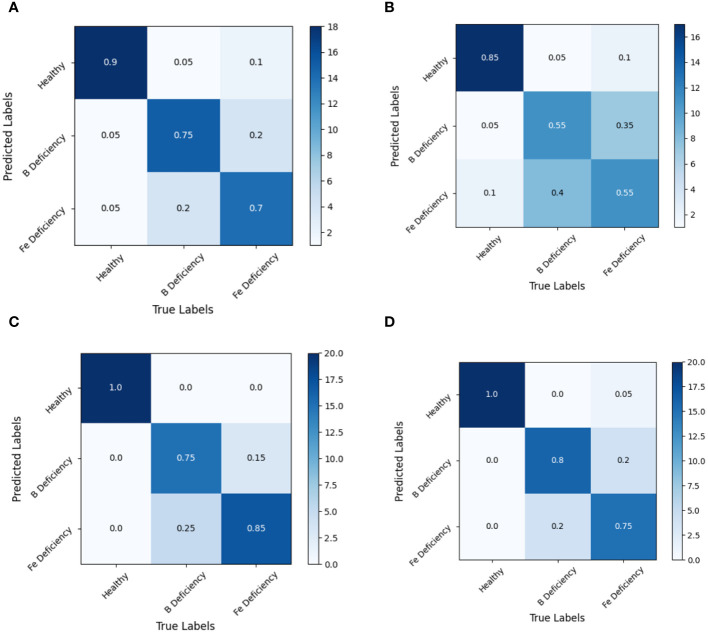
Discrimination results based on SVM model under different spectral pretreatments (Test set) for iron and boron deficiency in *Brassica napus*. **(A)** RAW, **(B)** 1st Der, **(C)** MSC, **(D)** 2nd Der.

By analyzing the confusion matrix of the modeling set (**Figure 5**) and the test set (**Figure 6**) based on the SVM model, it presented that the SVM model had a good spectral recognition effect for healthy and nutrient deficient rape leaves, with an accuracy rate of more than 90%. The recognition effect of iron and boron deficient rapeseed leaves is average, with the 1st Der data having the worst effect, with an accuracy rate of only 55%. The accuracy rates of RAW, MSC, and 2nd Der data are all between 70% and 85%.

Experiments also shown that SVM models based on full band spectral data can effectively identify healthy rapeseed leaves and rapeseed leaves lacking iron and boron elements. However, the recognition accuracy between iron deficient and boron deficient leaves still needed to be improved.

**Figure 7:**
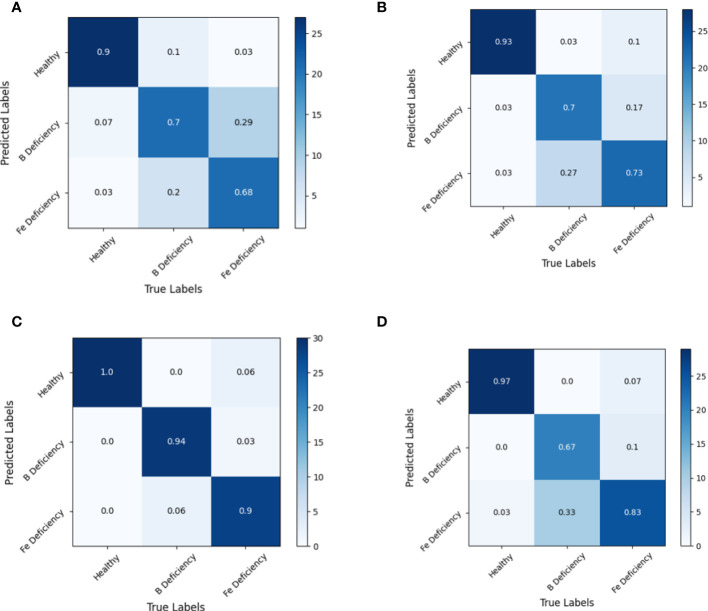
Discrimination results based on LDA models under different spectral pretreatments (Modeling set) for iron and boron deficiency in *Brassica napus*. **(A)** RAW, **(B)** 1st Der, **(C)** MSC, **(D)** 2nd Der.

**Figure 8:**
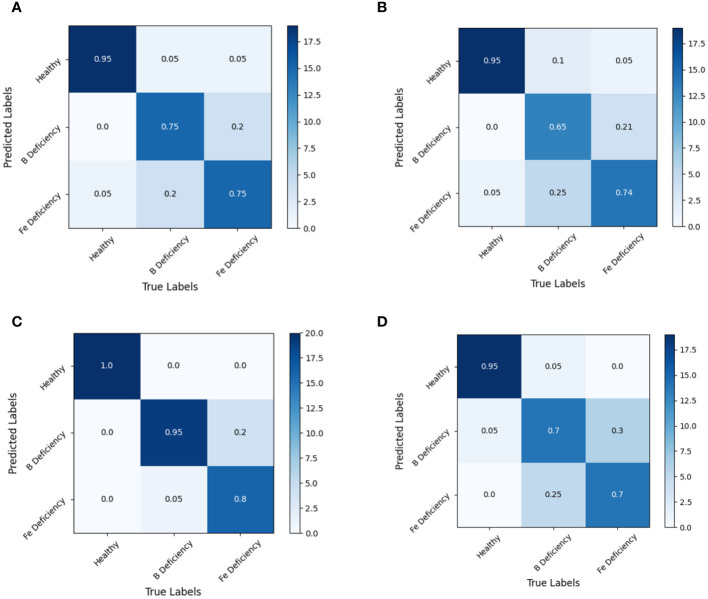
Discrimination results based on LDA model under different spectral pretreatment (test set). **(A)** RAW, **(B)** 1st Der, **(C)** MSC, **(D)** 2nd Der.

The analysis of the confusion matrix of the modeling set (**Figure 7**) and the test set (**Figure 8**) based on the LDA model showed that the LDA model was superior to the SVM model in spectral recognition of healthy and nutrient deficient rapeseed leave. Its accuracy in the test set is more than 95%. In the recognition of iron and boron deficient rapeseed leaves, MSC data showed significantly better performance than RAW, 1st Der, and 2nd Der data, with average accuracy exceeding 90% in both training and testing sets.

Further analysis of the discrimination results of the MSC-LDA model showed that both the modeling and prediction sets had a 100% accuracy in discriminating normal samples. In general, the discrimination accuracy of samples with boron deficiency was higher than that of samples with iron deficiency. From the confusion matrix, two samples with boron deficiency in the modeling set were wrongly identified as samples with iron deficiency symptoms, while one sample with iron deficiency symptoms was wrongly identified as samples with boron deficiency, and two samples are wrongly identified as healthy samples. The prediction set discrimination results also showed that one sample with boron deficiency was wrongly identified as samples with iron deficiency symptoms, and four samples with iron deficiency symptoms were wrongly identified as samples with iron deficiency.

The results of this experiment indicate that the MSC-LDA model achieved the highest accuracy in the combination of data preprocessing and modeling methods for Brassica napus iron and boron. The overall discrimination accuracy of the modeling set reached 96.67%, and the overall discrimination accuracy of the prediction set reached 91.67%.

### 3.3 Feature band screening results

**Figure 9:**
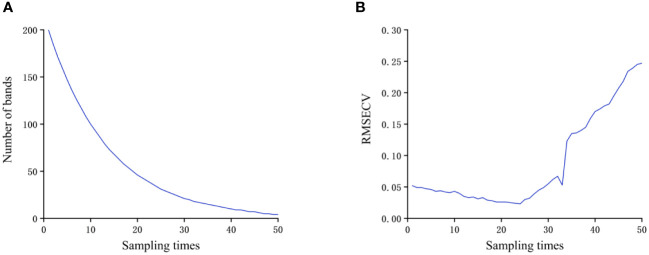
Impact of the number of bands involved in modeling on model accuracy. **(A)** Change trend of band number; **(B)** Model RMSECV change trend.

**Figure 9A** shows the process of reducing the number of bands involved in modeling through 50 Monte Carlo sampling (MC) of the sample data. **Figure 9B** shows the cross-validation error curve of the PLS model using the Leave on One Out (LOO) method as the number of bands involved in modeling decreases. From the above two curves, it could be seen that as the number of bands involved in modeling gradually decreases, the Root Mean Square Error of Cross Validation (RMSECV) of the model first shows a slow decreasing trend. It indicated that there is indeed a lot of redundant information in the spectral raw data containing more than 600 bands. Screening out certain band data could not only reduce computational complexity, but also improve the accuracy of the model to a certain extent. When the sampling frequency starts from 24, the RMSECV of the model in the training set gradually increased as the number of modeling bands decreases, indicating that some band data useful for classification modeling begins to be eliminated.

The above phenomenon indicates that there is indeed a large amount of redundant information in the original spectrum that is useless for the classification and recognition of iron and boron stress in rapeseed. It is meaningful to reduce the dimensionality of the original spectral data.

SPA and CARS were used to reduce the dimensionality of rapeseed leaf spectral data, as shown in **Table 3**. A total of 18 characteristic wavelengths were selected by SPA and defined as subset 1. CARS screened a total of 33 wavelengths and defined them as subset 2.

**Table 3:** Selected characteristic wavebands by SPA and CARS.

Data name	Wavelength(nm)
Dataset 1	410, 411, 416, 421, 426, 429, 434, 437, 441, 442, 672, 691, 722, 737, 981, 990, 997
Dataset 2	401, 402, 405, 406, 408, 411, 412, 414, 417, 418, 422, 426, 427, 429, 430, 434, 438, 445, 446, 449, 453, 455, 674, 688, 812, 864, 882, 919, 955, 973, 977, 980, 992

### 3.4 Establishment of a deficiency recognition and classification model based on feature band information

**Figure 10:**
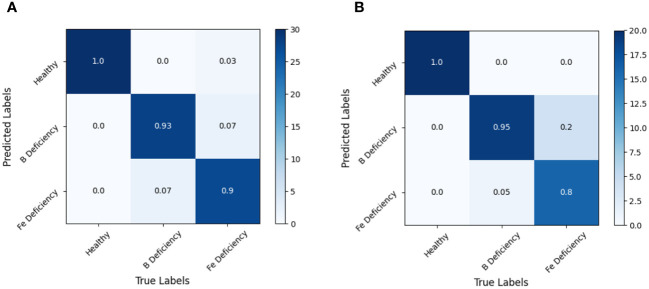
MSC-SPA-LDA model discrimination result confusion matrix. **(A)** training set, **(B)** test set.

**Figure 11:**
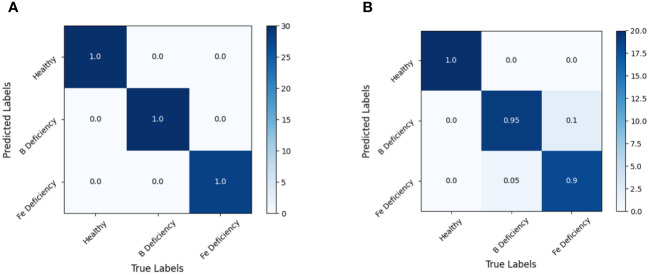
MSC-CARS-LDA model discrimination result confusion matrix. **(A)** training set, **(B)** test set.

Since the MSC-LDA model is superior to other discriminative model when full band spectral information modeling is used, the MSC-SPA-LDA and MSC-CARS-LDA modeling and discrimination are conducted using two characteristic wavelength subsets screened according to SPA and CARS. The prediction results are shown in **Table 4**, and the confusion matrix is shown in **Figure 10** (MSC-SPA-LDA) and **Figure 11** (MSC-CARS-LDA).

**Table 4:** MSC-LDA discrimination model based on characteristic wavelength.

Model	Training Set		Test Set	
	Nc/Nt	Accuracy	Nc/Nt	Accuracy
MSC-SPA-LDA	85/90	94.44%	55/60	91.67%
MSC-CARS-LDA	90/90	100.00%	57/60	95.00%

Analysis of the discrimination results of the MSC-SPA-LDA model showed that the accuracy of the MSC-SPA-LDA model based on SPA feature bands is 94.44% on the training set and 91.67% on the test set, which is slightly lower than the MSC-LDA model based on full band. However, due to its significant reduction in the number of input variables in the model, the running speed of the model is significantly improved, and the accuracy is within an acceptable range, so the MSC-SPA-LDA model has better applicability than the MSC-LDA model.

By analyzing the discrimination results of the MSC-CARS-LDA model, it could be concluded that the MSC-CARS-LDA model based on CARS feature bands achieved 100% and 95% accuracy on the training and testing sets, respectively, making it the model with the highest recognition accuracy in this experiment.

The feature wavelengths of subset 2 selected based on the CARS algorithm were mainly concentrated between the regions of 400–450 nm and 800–1000 nm, especially in the blue-violet light region of 400–450 nm, which is not the green peak region with the greatest difference in the 500–650 nm spectral curve. This indicates that the degree of leaf chlorosis is not the only basis for discrimination in this recognition system.

## 4 Discussion

The deficiency of iron and boron could lead to a decrease in chlorophyll content in the leaves, weakening their absorption of solar radiation, and causing an overall increase in the spectral reflectance of the leaves in the wavelength range of 400–700 nm, resulting in a “blue shift” phenomenon at the “red edge” position. This was consistent with previous research results (Yang et al., 2018). Considering the “green peak” at 550 nm, the spectral reflectance difference was the largest. The increase in green peak caused by iron deficiency was more intense than that caused by boron deficiency, indicating that the level of plant nutrient element content was closely related to spectral characteristics. When the plants were in deficiency of iron or boron, the total chlorophyll content of their leaves might reduce. That would lead to weak absorption of solar radiation and an increase in the reflectance and transmittance of incident light, which has been proven in crops like sorghum and sugar beet (Teixeira et al., 2020; Wu et al., 2021). The symptoms of nutrient deficiency in rapeseed leaves appeared because of the decrease of chlorophyll content, which might cause corresponding spectral responses such as an increase in green peaks. This provides a basis for conducting spectral recognition and identification.

The spectral reflectance of plant leaves in the range of 400–1000 nm indicated spectral responses to various factors such as plant metabolites, chlorophyll, water content, internal structure of leaf surfaces, and physical properties of plant leaves. The correlation between spectral reflectance of different bands and the abundance or deficiency of iron and boron elements in rapeseed plants was comprehensive responses of the rapeseed plants to the nutrients status and environment, rather than the direct correlation between spectral values and iron and boron content. Therefore, machine learning algorithms was employed in this study for further analysis. Two pattern recognition methods, LDA and SVM, were used to identify different deficiency symptoms. The LDA algorithm achieves better recognition results, and the CARS algorithm performs better than the SPA algorithm in feature wavelength screening. Through the analysis of confusion matrix, it presented that the recognition rate of the recognition model established in this study for healthy rape leaves was always higher than 90%. The recognition of iron deficient leaves and boron deficient leaves presented some confusion of samples. From **Figure 5**, it could also be seen that the green peak increase response caused by iron deficiency was stronger than that caused by boron deficiency. It might suggest that the physiological response to spectral properties from iron deficiency was more sensitive than that from boron deficiency in rapeseed plants (Sarafi et al., 2018).

## 5 Conclusion

The spectral response of normal, iron deficient, and boron deficient rapeseed plants was investigated using hyperspectral imaging technology in this study. Thus, it could conclude that, (1) with employing spectral imaging technology, it is capable to identify the iron and boron deficiency symptoms in rapeseed from healthy plants at early growth stage based on full band and featured band; (2) the LDA discriminative model established by screening characteristic wavelengths could be optimized using CARS for further field application with lower data consumption and faster calculation, and the recognition accuracy of its modeling set and prediction set could be 92.22% and 86.67%.

## Data availability statement

The original contributions presented in the study are included in the article/supplementary material. Further inquiries can be directed to the corresponding author.

## Author contributions

HL: Data curation, Funding acquisition, Investigation, Software, Writing – original draft, Writing – review & editing. LW: Data curation, Investigation, Writing – original draft, Writing – review & editing. CL: Resources, Writing – review & editing. LHW: Resources, Supervision, Writing – review & editing. SZ: Methodology, Resources, Supervision, Validation, Writing – review & editing. XC: Methodology, Resources, Supervision, Writing – review & editing. PW: Data curation, Funding acquisition, Investigation, Methodology, Software, Validation, Writing – original draft, Writing – review & editing.
